# Better response to Tanreqing injection in frequent acute exacerbation of chronic obstructive pulmonary disease (AECOPD) patients—Real-world evidence from a nationwide registry (ACURE) study

**DOI:** 10.3389/fphar.2023.1118143

**Published:** 2023-03-16

**Authors:** Guohui Fan, Dingyi Wang, Sinan Wu, Demin Li, Xiaoxia Ren, Fen Dong, Kewu Huang, Yahong Chen, Hongchun Zhang, Chen Wang, Ting Yang

**Affiliations:** ^1^ Department of Clinical Research and Data Management, Center of Respiratory Medicine, China-Japan Friendship Hospital, National Center for Respiratory Medicine, Institute of Respiratory Medicine, Chinese Academy of Medical Sciences, National Clinical Research Center for Respiratory Diseases, Beijing, China; ^2^ Department of Traditional Chinese Medicine for Pulmonary Diseases, Center of Respiratory Medicine, China-Japan Friendship Hospital, National Center for Respiratory Medicine, Institute of Respiratory Medicine, Chinese Academy of Medical Sciences, National Clinical Research Center for Respiratory Diseases, Beijing, China; ^3^ Department of Pulmonary and Critical Care Medicine, Center of Respiratory Medicine, China-Japan Friendship Hospital, National Center for Respiratory Medicine, Institute of Respiratory Medicine, Chinese Academy of Medical Sciences, National Clinical Research Center for Respiratory Diseases, Beijing, China; ^4^ Beijing Key Laboratory of Respiratory and Pulmonary Circulation Disorders, Department of Pulmonary and Critical Care Medicine, Beijing Chao-Yang Hospital, Capital Medical University, Beijing Institute of Respiratory Medicine, Beijing, China; ^5^ Department of Respiratory and Critical Care Medicine, Peking University Third Hospital, Beijing, China

**Keywords:** Tanreqing injection, frequent AECOPD, comparative study, real-world, efficacy

## Abstract

**Objective:** Our aim was to systematically investigate the efficacy of Tanreqing (TRQ) injection on in-hospital outcomes among inpatients with frequent or infrequent AECOPD.

**Methods:** In this ongoing, nationwide multicenter registry designed to investigate clinical characteristics, management, and prognoses of Chinese patients admitted for AECOPD in real-world settings, we collected characteristics, comorbidities, in-hospital prognoses, and information on the COPD assessment test (CAT) questionnaire, PEACE questionnaire, and modified British Medical Research Council (mMRC) questionnaire from each enrolled patient. Frequent AECOPD was determined as being admitted to the hospital ≥1 time or visiting the emergency room (ER) ≥ 2 times due to AECOPD within a year. A propensity match method and univariable and multivariable regression models were performed to analyze the efficacy of TRQ on clinical outcomes for inpatients with frequent AECOPD.

**Results:** A total of 4135 inpatients were involved in the analysis, including 868 administered with TRQ and 3267 not administered with TRQ. After propensity score match, among those administered with TRQ, 493 had frequent AECOPD and 358 had infrequent AECOPD. A significant reduction of CAT score at discharge (TRQ median 12, IQR 8.0–16.0; non-TRQ median 13, IQR 9.0–18.0, *p* = 0.0297), a lower rate of ICU admission (TRQ 0.8% vs. non-TRQ 2.6%, *p* = 0.0191), and a shorter length of stay (LOS) (TRQ median 11, IQR 9.0–14.0; non-TRQ median 11, IQR 8.0–14.0, *p* = 0.004) were observed in the TRQ group, compared with the non-TRQ group among frequent AECOPD patients. In the subgroup analysis, for those with a PEACE score >7 on admission, TRQ contributed to a significantly lower CAT score at discharge (*p* = 0.0084) and a numerically lower ICU admission rate with a marginal statistical significance. Among those with phlegm-heat symptom complex on admission ≥2, a lower CAT score at discharge and a lower ICU admission were also observed in the TRQ group.

**Conclusion:** TRQ injection had better efficacy in patients with frequent AECOPD in reducing ICU admission and alleviating respiratory symptoms, especially for those with higher severity on admission or more phlegm-heat symptoms.

## Introduction

Chronic obstructive pulmonary disease (COPD) is characterized by chronic irreversible airflow limitation, and its high prevalence and mortality continue to lead to a heavy disease burden globally (GBD Chronic Respiratory Disease Collaborators, 2020; Global strategy for the diagnosis and management and prevention of chronic obstructive pulmonary disease, 2022). In China, the prevalence of COPD was 8.6%, and it was estimated that there were nearly 100 million patients among those over 20 years old in 2015.

Acute exacerbation of COPD (AECOPD) is an acute event where a worsening of respiratory symptoms beyond normal daily variations occurs, resulting in the need for a change in therapy (Wang et al., 2018). AECOPD is thought to be caused by complex interactions between the human body, pathogens, and the external environment, which leads to an increase in the inflammatory burden, and it is associated with increased airway and systemic inflammation and physiological changes (Wedzicha and Terence, 2007). Frequent exacerbation increases hospitalization and promotes disease progression, thus negatively impacting the management of COPD. A large cohort study revealed that AECOPD frequency in a single year predicts long-term AECOPD rate. Increasing frequency and severity of AECOPD is associated with the risk of death (Rothnie et al., 2018). The GOLD report uses a threshold of two or more acute exacerbations in the previous year, or at least one hospital admission related to acute exacerbation to identify individuals at a high risk of future events (Ouaalaya et al., 2020). Patients who have frequent exacerbations have higher mortality, worse quality of life, more future exacerbation events, and faster FEV1 decline than those with infrequent exacerbations (0–1/time per year) (Seemungal et al., 2000; Wedzicha et al., 2013; Mullerova et al., 2014). These patients also have an increased airway inflammation, which contributes to a higher risk of hospital admission and disease progression (Seemungal et al., 2000).

The management of AECOPD includes short-acting inhaled bronchodilators, systemic corticosteroids, antibiotics, oxygen therapy, and mechanical ventilation if required (Ko et al., 2016). Some clinical trials have evaluated the efficacy and safety of Chinese medicine injections for AECOPD patients and proved their effectiveness in inhibiting inflammation, regulating immune function, and alleviating symptoms (Li et al., 2010; Hu et al., 2021). Tanreqing (TRQ), an injectable prescription from traditional Chinese medicine with functions of clearing the heart, detoxifying, and resolving phlegm has been approved to treat acute respiratory infection (National Medical Products Administration, China, Number Z20030054). Several randomized clinical trials (RCTs) with limited sample sizes demonstrated the efficacy of TRQ for the treatment of AECOPD and severe pneumonia (Wang et al., 2020; Hu et al., 2021; Chen et al., 2022). However, the effectiveness of TRQ on frequent AECOPD in real-world applications has never been discussed. In our study, we conducted an analysis among AECOPD inpatients who were prescribed TRQ injection using the propensity score match (PSM) method, compared with those who did not use TRQ, to systematically investigate its efficacy on in-hospital outcomes of frequent or infrequent AECOPD.

## Materials and methods

### Study design and participants

Our study analyzed data from the acute exacerbation of chronic obstructive pulmonary disease inpatient registry (ACURE) study. The ACURE study is an ongoing, nationwide multicenter registry designed to investigate clinical characteristics, management, and prognoses of Chinese patients admitted for AECOPD in real-world settings (ClinicalTrials. gov identifier: NCT02657525). It started on 1 September 2017 and planned to recruit 7600 in-hospital AECOPD patients with a 3-year follow-up. The protocol and phase 1 results of the registry have been previously described (Pei et al., 2020; Liang et al., 2021). The study was approved by the ethics committee of China-Japan Friendship Hospital (No. 2015-88) and informed consent was obtained from all involved participants. The study was conducted in accordance with the Declaration of Helsinki.

## Measurements and outcomes

For each patient, a baseline survey was conducted within 1–3 days after hospitalization to collect information on medical history, physical examination, and inpatient diagnosis. During the hospital stay, the COPD assessment test (CAT) questionnaire, PEACE questionnaire (consisting of eight questions assessing daily variance of COPD symptoms, i.e., dyspnea, purulent sputum, sputum volume, upper respiratory tract infection, fever, wheezing, cough, and breath rate), modified British Medical Research Council (mMRC) questionnaire, medical examinations, laboratory tests, and treatments were recorded. Comorbidities including respiratory diseases, cardiovascular diseases, metabolic diseases (diabetes and osteoporosis), and digestive diseases, as well as malignancies other than lung cancer, peripheral arterial disease, venous thromboembolism, cerebrovascular disease, anxiety/depression, musculoskeletal dysfunction, chronic kidney disease, etc., were recorded.

During hospitalization, treatment (including the application of TRQ) and auxiliary examination results including laboratory and lung function tests were recorded if available. All auxiliary examinations were conducted at local sites, and the results were uploaded to the database by investigators. If laboratory data were unavailable during hospitalization, the most recent results within 3 days before admission were used for imputation. If multiple tests were conducted after admission, the earliest one was used. The severity of airflow limitation was classified into four grades based on the 2017 Global Initiative for Chronic Obstructive Lung Disease (GOLD) report: GOLD 1 (forced expiratory volume in one second [FEV1]% predicted ≥80), GOLD 2 (50 ≤ FEV1% predicted <80), GOLD 3 (30 ≤ FEV1% predicted <50), and GOLD 4 (FEV1% predicted <30) (Global strategy for the diagnosis and management and prevention of chronic obstructive pulmonary disease, 2017). Total direct costs were calculated in US dollars using the average exchange rate in 2019 (one US dollar was equivalent to 6.90 yuan).

Patients were divided into “frequent AECOPD” if they were admitted to the hospital ≥1 time or visited the emergency room (ER) ≥ 2 times due to AECOPD within a year and “infrequent AECOPD” if they were never admitted to the hospital or visited the emergency room (ER) < 2 times due to AECOPD, according to the GOLD report (Global strategy for the diagnosis and management and prevention of chronic obstructive pulmonary disease, 2017). According to the theory of traditional Chinese medicine, patients were defined as having “phlegm and heat” syndrome if they had ≥ two of the following symptoms: fever, pharyngalgia, purulent sputum, and sputum over 50 mL/day. Those without a single of the above symptoms were defined as having “no phlegm and heat” syndrome.

The primary outcome was a PEACE score at discharge (Zheng et al., 2008). The secondary outcomes were ICU admission, the change of CAT score at discharge, mMRC dyspnea grade at discharge, length of hospitalization, and total cost of hospitalization.

### Statistical analysis

Baseline patient characteristics were expressed in terms of descriptive statistics. Categorical variables were summarized as frequency (percentage). Continuous variables were presented as mean (standard deviation, SD) or median (interquartile range, IQR). *p* values were calculated by students’ t-test, χ^2^ test, or Fisher exact test where appropriate.

A propensity score was estimated by logistic regression to determine the probability of TRQ treatment of each patient conditionally on observed covariates. Based on the propensity score, we performed 2:1 match-to-match patients who were not administered with TRQ to those who were on a range of ±0.0001 to ± 0.1. The match started with the range of ± 0.0001, and those who were matched were extracted from the database and excluded from the following ranges. If more than two patients who were not administered TRQ were detected, only two of them were selected randomly. Variables involved in the propensity score estimation included age, drug therapy, PEACE score at admission, mMRC score at admission, hospitalization frequency, diagnosis as COPD for the first time, cor pulmonale, non-drug therapy, cough frequency, expectoration, and fever. The propensity score matches were performed in the analyses between frequent AECOPD and infrequent AECOPD, PEACE score >7 and ≤7 on admission, phlegm-heat symptom complex ≥2 and <2 on admission, or phlegm-heat symptom complex ≥1 and <1 on admission. Univariable and multivariable analyses of the efficacy of TRQ on clinical outcomes for inpatients with frequent AECOPD were performed by the logistic regression model or general linear model to investigate the efficacy of TRQ. In the multivariable model, covariates including age, drug therapy, PEACE score at admission, mMRC score at admission, hospitalization frequency, diagnosis as COPD for the first time, cor pulmonale, non-drug therapy, cough frequency, expectoration, and fever were adjusted.

All tests were two-sided and were considered statistically significant at a *p*-value of <0.05. All analyses were performed using SAS 9.4 software (Cary, NC, United States).

## Results

### Baseline characteristics

**FIGURE 1:**
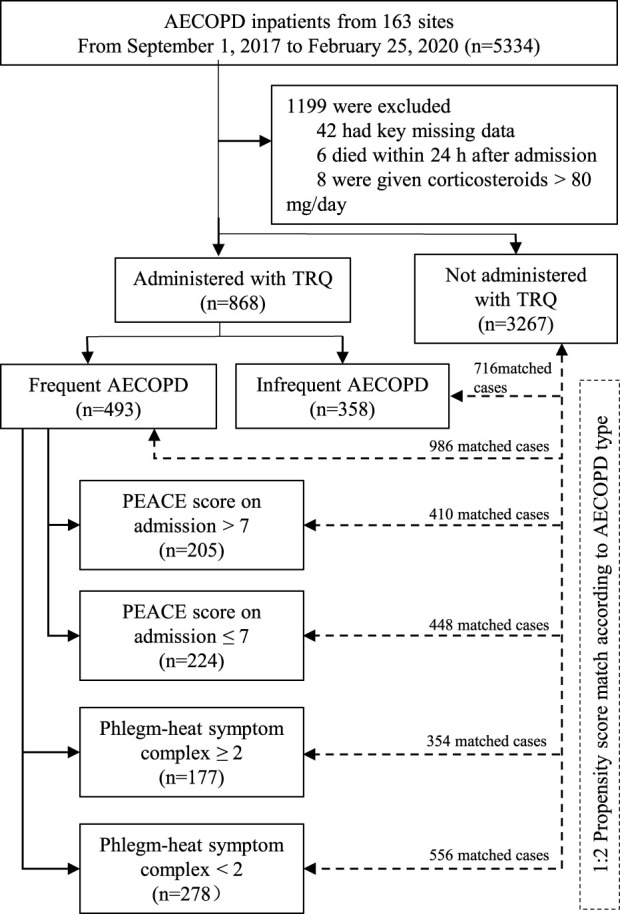
Flowchart of this study. Abbreviations: COPD, chronic obstructive pulmonary disease; AECOPD, acute exacerbation of COPD; TRQ, Tanreqing injection.

As is shown in the flow chart of this study, 5334 AECOPD inpatients were enrolled from 153 sites between 1 September 2017 and 25 February 2020. After excluding 1199 inpatients, 4135 inpatients were involved in the analysis, including 868 administered with TRQ and 3267 not administered with TRQ. After propensity score match, among those administered with TRQ, 493 had frequent AECOPD and 358 had infrequent AECOPD (Figure 1).

Among frequent AECOPD patients (n = 2179), those who have been prescribed TRQ (n = 505) had a significantly higher rate of cor pulmonale, cough, a larger amount of sputum, fever, and lower C-reactive protein (CRP) level on admission. The PEACE scores were higher among the TRQ group, compared with the non-TRQ group (TRQ median 8.0, interquartile range [IQR] 6.0–9.0 vs. non-TRQ median 7.0, IQR 6.0–9.0, *p* = 0.0038), indicating that those injected with TRQ had more severe symptoms on admission. Similar results were revealed among the infrequent AECOPD patients (Table 1). After the propensity match, the demographic and baseline characteristics were balanced between TRQ and non-TRQ groups in patients with frequent AECOPD or infrequent AECOPD (Supplementary Table S1).

**TABLE 1:** Characteristics of inpatients with frequent AECOPD or Infrequent AECOPD before propensity score match.

Frequent AECOPD					Infrequent AECOPD			
Characteristics	Total	TRQ	Without TRQ	p	Total	TRQ	Without TRQ	p
	N = 2179	N = 505	N = 1674		N = 1956	N = 363	N = 1593	
Age, years	70.8 (64.8, 77.5)	71.0 (65.1, 77.1)	70.6 (64.7, 77.6)	0.788	69.3 (62.8, 76.0)	69.6 (62.9, 75.6)	69.2 (62.7, 76.1)	0.7567
Male	1747 (80.2)	397 (78.6)	1350 (80.6)	0.3156	1425 (72.9)	277 (76.3)	1148 (72.1)	0.1009
BMI, Kg/m^2^	21.8 (19.5, 24.2)	21.8 (19.4, 24.2)	21.8 (19.5, 24.2)	0.9525	22.0 (19.7, 24.5)	22.0 (19.6, 24.2)	22.0 (19.8, 24.6)	0.1882
Smoking				0.9971				0.6736
Current smoking	406 (18.6)	94 (18.6)	312 (18.6)		556 (28.4)	105 (28.9)	451 (28.3)	
Never smoking	706 (32.4)	163 (32.3)	543 (32.4)		685 (35.0)	120 (33.1)	565 (35.5)	
Quit smoking	1067 (49.0)	248 (49.1)	819 (48.9)		715 (36.6)	138 (38.0)	577 (36.2)	
Pulmonary thromboembolism	10 (0.5)	3 (0.6)	7 (0.4)	0.6189	4 (0.2)	0 (0.0)	4 (0.3)	0.1998
Pulmonary artery hypertension	99 (4.5)	28 (5.5)	71 (4.2)	0.2177	41 (2.1)	8 (2.2)	33 (2.1)	0.8738
Hypertension	720 (33.0)	160 (31.7)	560 (33.5)	0.4587	628 (32.1)	106 (29.2)	522 (32.8)	0.1889
Myocardial infarction	367 (16.8)	81 (16.0)	286 (17.1)	0.5822	300 (15.3)	44 (12.1)	256 (16.1)	0.0595
Cor pulmonale	525 (24.1)	143 (28.3)	382 (22.8)	0.0113	243 (12.4)	50 (13.8)	193 (12.1)	0.3873
Bronchiectasia	189 (8.7)	54 (10.7)	135 (8.1)	0.0658	131 (6.7)	24 (6.6)	107 (6.7)	0.9423
Non-drug therapy	1036 (47.5)	270 (53.5)	766 (45.8)	0.0024	451 (23.1)	91 (25.1)	360 (22.6)	0.3133
Drug therapy	1492 (68.5)	349 (69.1)	1143 (68.3)	0.7252	689 (35.2)	134 (36.9)	555 (34.8)	0.4552
Regular inhaled corticosteroid	100/178 (56.2)	35/61 (57.4)	65/117 (55.6)	0.8162	28/53 (52.8)	3/8 (37.5)	25/45 (55.6)	0.3449
Regular oral corticosteroid	24/94 (25.5)	8/33 (24.2)	16/61 (26.2)	0.833	6/40 (15.0)	4/11 (36.4)	2/29 (6.9)	0.0278
Inhaled corticosteroids	178 (8.2)	61 (12.1)	117 (7.0)	0.0003	53 (2.7)	8 (2.2)	45 (2.8)	0.5108
Inhaled bronchial dilator	444 (20.4)	125 (24.8)	319 (19.1)	0.0053	179 (9.2)	37 (10.2)	142 (8.9)	0.4457
PEACE at admission	7.0 (6.0, 9.0)	8.0 (6.0, 9.0)	7.0 (6.0, 9.0)	0.0038	7.0 (5.0, 9.0)	7.0 (6.0, 9.0)	7.0 (5.0, 8.0)	<.0001
CAT at admission	21.0 (16.0, 26.0)	21.0 (17.0, 25.0)	21.0 (16.0, 26.0)	0.924	19.0 (14.0, 24.0)	19.0 (15.0, 24.0)	19.0 (14.0, 24.0)	0.2091
mMRC at admission	3.0 (2.0, 3.0)	3.0 (2.0, 3.0)	3.0 (2.0, 3.0)	0.8024	3.0 (2.0, 3.0)	3.0 (2.0, 3.0)	3.0 (2.0, 3.0)	0.0166
Diagnosed with COPD for the first time	147 (6.7)	21 (4.2)	126 (7.5)	0.0082	1127 (57.6)	190 (52.3)	937 (58.8)	0.0242
Hospitalization frequency due to AECOPD	2.0 (1.0, 2.0)	2.0 (1.0, 2.0)	2.0 (1.0, 2.0)	0.5266	0.0 (0.0, 0.0)	0.0 (0.0, 0.0)	0.0 (0.0, 0.0)	1.000
Cough frequency				0.0084				0.0324
All day	1074 (49.3)	241 (47.7)	833 (49.8)		962 (49.2)	195 (53.7)	767 (48.1)	
Continuously	256 (11.7)	75 (14.9)	181 (10.8)		255 (13.0)	53 (14.6)	202 (12.7)	
No cough	35 (1.6)	2 (0.4)	33 (2.0)		39 (2.0)	3 (0.8)	36 (2.3)	
Occasionally	814 (37.4)	187 (37.0)	627 (37.5)		700 (35.8)	112 (30.9)	588 (36.9)	
Amount of sputum				0.046				0.0036
<50 mL	1300 (59.7)	282 (55.8)	1018 (60.8)		1146 (58.6)	188 (51.8)	958 (60.1)	
≥50 mL	879 (40.3)	223 (44.2)	656 (39.2)		810 (41.4)	175 (48.2)	635 (39.9)	
Purulent sputum	1069 (49.1)	258 (51.1)	811 (48.4)	0.2978	837 (42.8)	158 (43.5)	679 (42.6)	0.7539
Fever	347 (15.9)	101 (20.0)	246 (14.7)	0.0043	278 (14.2)	65 (17.9)	213 (13.4)	0.0255
Pharyngalgia	181 (8.3)	50 (9.9)	131 (7.8)	0.1385	140 (7.2)	26 (7.2)	114 (7.2)	0.9967
pH	7.4 (7.4, 7.4)	7.4 (7.4, 7.4)	7.4 (7.4, 7.4)	0.0038	7.4 (7.4, 7.4)	7.4 (7.4, 7.4)	7.4 (7.4, 7.4)	0.1243
PaO_2_ (mmHg)	43.5 (38.0, 52.0)	43.7 (38.0, 52.0)	43.0 (38.1, 51.0)	0.6548	42.0 (37.6, 48.0)	42.0 (37.8, 48.0)	42.0 (37.5, 48.0)	0.7088
PaO_2_ (mmHg)	73.0 (61.6, 89.6)	73.0 (61.8, 87.8)	75.0 (61.0, 94.0)	0.0651	72.1 (63.0, 85.9)	74.0 (64.2, 88.0)	72.0 (62.8, 85.0)	0.0745
High sensitivity C reactive protein, mg/dl	5.0 (1.2, 15.3)	5.0 (1.3, 16.0)	4.4 (0.8, 13.1)	0.0366	3.8 (0.9, 11.6)	2.6 (0.6, 10.0)	4.1 (1.0, 11.8)	0.0601
≥3, mg/dl	795/1297 (61.3)	608/965 (63.0)	187/332 (56.3)	0.0311	666/1220 (54.6)	111/231 (48.1)	555/989 (56.1)	0.0266
PCT, ng/ml	0.1 (0.0, 0.1)	0.1 (0.0, 0.1)	0.1 (0.0, 0.1)	0.3479	0.1 (0.0, 0.1)	0.1 (0.0, 0.1)	0.1 (0.0, 0.1)	0.7028
≥0.1, ng/ml	480/1266 (37.9)	351/952 (36.9)	129/314 (41.1)	0.1821	438/1173 (37.3)	76/225 (33.8)	362/948 (38.2)	0.2191
White blood cell count, × 10^9^/L	7.2 (5.7, 9.7)	7.2 (5.6, 9.7)	7.5 (5.8, 9.9)	0.0559	7.2 (5.7, 9.2)	7.1 (5.8, 9.2)	7.2 (5.7, 9.2)	0.9683
Neutrophils, %	72.0 (63.0, 80.3)	71.8 (63.0, 80.1)	73.0 (63.3, 80.9)	0.2839	69.1 (60.0, 78.3)	70.6 (59.9, 78.5)	69.0 (60.0, 78.3)	0.4635
Lymphocyte, %	16.8 (10.2, 24.0)	17.0 (10.3, 24.0)	16.4 (9.7, 23.8)	0.3638	18.7 (11.3, 27.0)	18.2 (11.1, 26.9)	19.0 (11.3, 27.0)	0.3586
Aspartate aminotransferase, U/L	19.1 (15.4, 26.0)	19.1 (15.4, 26.0)	19.1 (15.2, 26.0)	0.8056	20.0 (15.8, 26.0)	19.0 (15.0, 24.0)	20.0 (16.0, 26.4)	0.0093
>40, U/L	169/2067 (8.2)	122/1572 (7.8)	47/495 (9.5)	0.2195	129/1886 (6.8)	19/352 (5.4)	110/1534 (7.2)	0.2346

Note. Data were expressed as n (%) or median (interquartile range), where appropriate. *p* values were calculated by the Mann-Whitney *U* test, Chi-square test, or Fisher exact test, where appropriate. Abbreviations: COPD, chronic obstructive pulmonary disease; AECOPD, acute exacerbation of COPD; TRQ, tanreqing injection; BMI, body mass index; CAT, the COPD assessment test; mMRC, modified British medical research council; PCT, procalcitonin.

### Treatment and clinical outcomes after PSM

After the propensity score match, a significant reduction of CAT score at discharge (TRQ median 12, IQR 8.0–16.0; non-TRQ median 13, IQR 9.0–18.0, *p* = 0.0297), a lower rate of ICU admission (TRQ 0.8% Vs. non-TRQ 2.6%, *p* = 0.0191), and a shorter length of stay (LOS) (TRQ median 11 days, IQR 9.0–14.0; non-TRQ median 11 days, IQR 8.0–14.0, *p* = 0.004) were observed in the TRQ group, compared with the non-TRQ group among frequent AECOPD patients. Treatment and clinical outcomes including PEACE at discharge, PEACE difference, mMRC at discharge, mMRC difference, death or worsened cases, cost, antibiotics, and systemic corticosteroid were compared between TRQ and non-TRQ groups among frequent AECOPD patients. No significant difference in outcomes was shown in infrequent AECOPD patients (Table 2). After adjusting for covariates, the TRQ group was independently associated with a lower CAT score at discharge (β −0.90, 95% confidence interval [95% CI] −1.53 to −0.27, *p* = 0.0050) and lower ICU admission (odds ratio [OR] 0.30, 95% CI 0.10–0.87, *p* = 0.0050) (Table 3).

**TABLE 2:** Treatment and clinical outcomes of inpatients with frequent AECOPD or Infrequent AECOPD after propensity score match.

Frequent AECOPD					Infrequent AECOPD			
Characteristics	Total	TRQ	Without TRQ	p	Total	TRQ	Without TRQ	p
	N = 1479	N = 493	N = 986		N = 1074	N = 358	N = 716	
PEACE at discharge	3.0 (2.0, 4.0)	3.0 (2.0, 4.0)	3.0 (2.0, 4.0)	0.4074	3.0 (2.0, 4.0)	3.0 (2.0, 4.0)	3.0 (2.0, 4.0)	0.6681
PEACE difference	−4.0 (−6.0, −3.0)	−4.0 (−6.0, −3.0)	−4.0 (−6.0, −3.0)	0.321	−5.0 (−6.0, −3.0)	−5.0 (−6.0, −3.0)	−5.0 (−6.0, −3.0)	0.3576
CAT at discharge	13.0 (9.0, 17.0)	12.0 (8.0, 16.0)	13.0 (9.0, 18.0)	0.0297	10.0 (8.0, 14.0)	10.0 (7.5, 14.0)	10.0 (8.0, 15.0)	0.3198
mMRC at discharge	2.0 (1.0, 2.0)	2.0 (1.0, 2.0)	2.0 (1.0, 2.0)	0.2772	1.0 (1.0, 2.0)	1.0 (1.0, 2.0)	1.0 (1.0, 2.0)	0.7438
mMRC difference	−1.0 (−2.0, 0.0)	−1.0 (−2.0, 0.0)	−1.0 (−2.0, 0.0)	0.5614	−1.0 (−2.0, −1.0)	−1.0 (−2.0, 0.0)	−1.0 (−2.0, −1.0)	0.9162
length of hospital stay, days	11.0 (9.0, 14.0)	11.0 (9.0, 14.0)	11.0 (8.0, 14.0)	0.004	10.0 (8.0, 13.0)	10.0 (8.0, 13.0)	10.0 (8.0, 12.0)	0.2043
Death or worsening in the hospital	8 (0.5)	2 (0.4)	6 (0.6)	0.6074	2 (0.2)	1 (0.3)	1 (0.1)	0.6271
ICU admission	30/1478 (2.0)	4/492 (0.8)	26/986 (2.6)	0.0191	17 (1.6)	3 (0.8)	14 (2.0)	0.1667
Oxygen support				0.7970				0.9644
No oxygen support	169 (11.4)	58 (11.8)	111 (11.3)		240 (22.3)	80 (22.3)	160 (22.3)	
Tube/mask	1129 (76.3)	382 (77.5)	747 (75.8)		773 (72.0)	259 (72.3)	514 (71.8)	
HFNC	15 (1.0)	4 (0.8)	11 (1.1)		5 (0.5)	1 (0.3)	4 (0.6)	
NPPV	160 (10.8)	47 (9.5)	113 (11.5)		52 (4.8)	17 (4.7)	35 (4.9)	
IPPV	6 (0.4)	2 (0.4)	4 (0.4)		4 (0.4)	1 (0.3)	3 (0.4)	
Total cost of hospitalization (USD)	1504.9 (1078.1, 2118.2)	1459 (1060.9, 2022.2)	1517.3 (1087, 2175.2)	0.0821	1350.6 (985.4, 1956.8)	1337.6 (990.1, 2000.1)	1359.4 (976.4, 1950.2)	0.9473
Antibiotics	1378/1478 (93.2)	465/493 (94.3)	913/985 (92.7)	0.2394	1002/1073 (93.4)	347/358 (96.9)	655/715 (91.6)	0.001
Systemic Corticosteroid	1192/1478 (80.6)	391/493 (79.3)	801/985 (81.3)	0.3565	834/1073 (77.7)	275/358 (76.8)	559/715 (78.2)	0.6121

Note. The exchange rate of RMB was 6.9 yuan to the US dollar. Data were expressed as n (%) or median (interquartile range), where appropriate. *p* values were calculated by the Mann-Whitney *U* test, Chi-square test, or Fisher exact test, where appropriate. Abbreviations: COPD, chronic obstructive pulmonary disease; AECOPD, acute exacerbation of COPD; TRQ, tanreqing injection; CAT, the COPD assessment test; mMRC, modified British medical research council; ICU, intensive care unit; HFNC, high-flow nasal cannula oxygen therapy; NPPV, non-invasive positive pressure ventilation; IPPV, invasive positive pressure ventilation.

**TABLE 3:** Univariable and multivariable analyses of the efficacy of TRQ on clinical outcomes for inpatients with frequent AECOPD.

		Univariable				Multivariable			
Outcomes		OR/β (95% CI)			P	OR/β (95% CI)			P
PEACE at discharge		−0.08 (−0.25,0.10)			0.3829	−0.08 (−0.23,0.08)			0.3532
CAT at discharge		−0.90 (−1.56,-0.25)			0.0070	−0.90 (−1.53,−0.27)			0.0050
mMRC at discharge		−0.06 (−0.17,0.04)			0.2381	−0.06 (−0.15,0.03)			0.1821
PEACE difference		−0.09 (−0.33,0.15)			0.4655	−0.08 (−0.23,0.08)			0.3532
mMRC difference		−0.06 (−0.17,0.04)			0.2152	−0.06 (−0.15,0.03)			0.1821
length of hospital stay (days, log-transformed)		0.04 (−0.02,0.09)			0.1701	0.04 (−0.01,0.09)			0.1577
Death or worsening in the hospital	Non-TRQ	Reference				Reference			
	TRQ	0.67 (0.13−3.31)			0.6185	0.74 (0.14−3.83)			0.7218
ICU admission	Non-TRQ	Reference				Reference			
	TRQ	0.30 (0.11−0.87)			0.0269	0.30 (0.10−0.87)			0.0267
NPPV	Non-Tanreqing	Ref				Ref			
	Tanreqing	0.81 (0.57−1.17)			0.2614	0.85 (0.58−1.24)			0.4005
Total cost of hospitalization (USD, log-transformed)		0.01 (−0.07,0.09)			0.8257	0.01 (−0.07,0.09)			0.7748

Note. The exchange rate of RMB was 6.9 yuan to the US dollar. OR (95% CI) and β (95% CI) were estimated by logistic regression models or general linear models, respectively. In the multivariable model, TRQ was adjusted for covariates including age, drug therapy, PEACE score at admission, mMRC score at admission, hospitalization frequency, diagnosis as COPD for the first time, cor pulmonale, non-drug therapy, cough frequency, expectoration, and fever.

Abbreviations: COPD, chronic obstructive pulmonary disease; AECOPD, acute exacerbation of COPD; TRQ, tanreqing injection; CAT, the COPD assessment test; mMRC, modified British medical research council; ICU, intensive care unit; NPPV, non-invasive positive pressure ventilation; OR, odds ratio; 95% CI, 95% confidence interval.

### Subgroup analysis after PSM

To further analyze the potential of susceptible patients treated with TRQ, a series of subgroup analyses were conducted among frequent AECOPD inpatients with PEACE scores >7 or ≤7 on admission and different phlegm-heat symptom complex. As shown in Table 4 and supplementary table 2, among those with PEACE score >7 on admission, TRQ contributed to a significantly lower CAT score at discharge (TRQ median 12.0, IQR 8.5–16.0; non-TRQ median 13.0, median 10.0–19.0, *p* = 0.0084) and numerically lower ICU admission rate with marginal statistical significance (TRQ 1.0%; non-TRQ 3.7%, *p* = 0.0557). Among those with phlegm-heat symptom complex on admission ≥2, lower CAT score at discharge and lower ICU admission were also observed in the TRQ group (*p* = 0.0145 and 0.0434, respectively); among those with phlegm-heat symptom complex on admission <2, TRQ contributed to the lower total cost of hospitalization (*p* = 0.0126) (Table 4). The treatment of TRQ may prolong the length of hospital stay in patients with phlegm-heat symptom complex on admission ≥1 (*p* = 0.0271) but still be effective in lowering CAT score at discharge among those without any phlegm-heat symptom complex (*p* = 0.0438) (Supplementary Table S2).

**TABLE 4:** Subgroup analyses on treatment and clinical outcomes of inpatients with frequent AECOPD after propensity match.

Characteristics	Total	TRQ	Without TRQ	p	Total	TRQ	Without TRQ	p
PEACE on admission	>7				≤7			
	N = 615	N = 205	N = 410		N = 672	N = 224	N = 448	
PEACE at discharge	4.0 (3.0, 4.0)	4.0 (3.0, 4.0)	4.0 (3.0, 4.0)	0.3084	3.0 (2.0, 4.0)	3.0 (2.0, 4.0)	3.0 (2.0, 4.0)	0.4652
CAT at discharge	13.0 (10.0, 18.0)	12.0 (8.5, 16.0)	13.0 (10.0, 19.0)	0.0084	12.0 (9.0, 17.0)	12.0 (8.0, 16.0)	12.0 (9.0, 17.0)	0.0921
PEACE difference	−6.0 (−7.0, −4.0)	−6.0 (−7.0, −4.0)	−5.0 (−7.0, −4.0)	0.5022	−3.0 (−4.0, −2.0)	−3.0 (−4.0, −2.0)	−3.0 (−4.0, −2.0)	0.4585
mMRC at discharge	2.0 (1.0, 2.0)	2.0 (1.0, 2.0)	2.0 (1.0, 2.0)	0.361	1.0 (1.0, 2.0)	1.0 (1.0, 2.0)	1.5 (1.0, 2.0)	0.3015
mMRC difference	−1.0 (−2.0, −1.0)	−1.0 (−2.0, −1.0)	−1.0 (−2.0, −1.0)	0.5721	−1.0 (−1.0, 0.0)	−1.0 (−1.0, 0.0)	−1.0 (−1.0, 0.0)	0.5021
length of hospital stay, days	12.0 (9.0, 15.0)	12.0 (10.0, 15.0)	12.0 (9.0, 15.0)	0.2145	10.0 (8.0, 13.0)	11.0 (9.0, 13.0)	10.0 (8.0, 13.0)	0.0112
Total cost of hospitalization (USD)	1652.8 (1175.9, 2406.4)	1607.0 (1140.9, 2211.8)	1690.7 (1197.3, 2530.5)	0.1570	1362.2 (1013.2, 1898.8)	1354.4 (996.3, 1738.3)	1366.4 (1017.2, 1993.2)	0.2520
Death or worsening in the hospital	6 (1.0)	1 (0.5)	5 (1.2)	0.3558	2 (0.3)	1 (0.4)	1 (0.2)	0.6269
ICU admission	17 (2.8)	2 (1.0)	15 (3.7)	0.0557	8/671 (1.2)	1/223 (0.4)	7/448 (1.6)	0.1739
Oxygen support				0.2748				0.2175
No oxygen support	58 (9.4)	20 (9.8)	38 (9.3)		96 (14.3)	30 (13.4)	66 (14.7)	
Tube/mask	474 (77.1)	163 (79.5)	311 (75.9)		507 (75.4)	166 (74.1)	341 (76.1)	
HFNC	8 (1.3)	1 (0.5)	7 (1.7)		4 (0.6)	2 (0.9)	2 (0.4)	
NPPV	72 (11.7)	21 (10.2)	51 (12.4)		63 (9.4)	24 (10.7)	39 (8.7)	
IPPV	3 (0.5)	0 (0.0)	3 (0.7)		2 (0.3)	2 (0.9)	0 (0.0)	
Antibiotics	587/614 (95.6)	194/205 (94.6)	393/409 (96.1)	0.4073	612 (91.1)	209 (93.3)	403 (90.0)	0.1513
Corticosteroid	511/614 (83.2)	168/205 (82.0)	343/409 (83.9)	0.5499	540 (80.4)	176 (78.6)	364 (81.3)	0.41

Phlegm-heat symptom complex on admission		≥2			<2			
	N = 531	N = 177	N = 354		N = 834	N = 278	N = 556	
PEACE at discharge	3.0 (2.0, 4.0)	3.0 (2.0, 4.0)	3.0 (3.0, 4.0)	0.3992	3.0 (2.0, 4.0)	3.0 (2.0, 4.0)	3.0 (2.0, 4.0)	0.6273
CAT at discharge	13.0 (9.0, 17.0)	12.0 (8.0, 16.0)	13.0 (9.0, 18.0)	0.0145	12.0 (9.0, 17.0)	12.0 (9.0, 17.0)	12.0 (9.0, 18.0)	0.6000
PEACE difference	−6.0 (−7.0, −4.0)	−6.0 (−7.0, −4.0)	−5.0 (−7.0, −4.0)	0.2391	−4.0 (−5.0, −2.0)	−4.0 (−5.0, −2.0)	−4.0 (−5.0, −2.0)	0.8013
mMRC at discharge	2.0 (1.0, 2.0)	2.0 (1.0, 2.0)	2.0 (1.0, 2.0)	0.0676	2.0 (1.0, 2.0)	2.0 (1.0, 2.0)	2.0 (1.0, 2.0)	0.5671
mMRC difference	−1.0 (−2.0, −1.0)	−1.0 (−2.0, −1.0)	−1.0 (−2.0, 0.0)	0.06	−1.0 (−2.0, 0.0)	−1.0 (−2.0, 0.0)	−1.0 (−2.0, 0.0)	0.4352
length of hospital stay, days	11.0 (9.0, 15.0)	12.0 (10.0, 15.0)	11.0 (9.0, 15.0)	0.3357	11.0 (9.0, 14.0)	11.0 (9.0, 14.0)	11.0 (8.0, 13.0)	0.0413
Total cost of hospitalization (USD)	1616.8 (1172.8, 2274.5)	1652.0 (1239.2, 2264.2)	1585.8 (1159.7, 2275.4)	0.5277	1415.3 (1011.3, 2013.3)	1331.5 (978.7, 1801.3)	1485.7 (1023.5, 2136.8)	0.0126
Death or worsening in the hospital	3 (0.6)	0 (0.0)	3 (0.8)	0.1182	6 (0.7)	2 (0.7)	4 (0.7)	1.0000
ICU admission	5 (0.9)	0 (0.0)	5 (1.4)	0.0434	23/833 (2.8)	4/277 (1.4)	19/556 (3.4)	0.1015
Oxygen support				0.5143				0.3974
No oxygen support	52 (9.8)	20 (11.3)	32 (9.0)		97 (11.6)	35 (12.6)	62 (11.2)	
Tube/mask	420 (79.1)	135 (76.3)	285 (80.5)		627 (75.2)	214 (77.0)	413 (74.3)	
HFNC	6 (1.1)	2 (1.1)	4 (1.1)		8 (1.0)	2 (0.7)	6 (1.1)	
NPPV	51 (9.6)	20 (11.3)	31 (8.8)		98 (11.8)	25 (9.0)	73 (13.1)	
IPPV	2 (0.4)	0 (0.0)	2 (0.6)		4 (0.5)	2 (0.7)	2 (0.4)	
Antibiotics	500/530 (94.3)	168/177 (94.9)	332/353 (94.1)	0.6847	766 (91.8)	263 (94.6)	503 (90.5)	0.0396
Corticosteroid	436/530 (82.3)	145/177 (81.9)	291/353 (82.4)	0.8835	673 (80.7)	219 (78.8)	454 (81.7)	0.3209

Note. The phlegm-heat symptom complex includes fever, pharyngalgia, expectoration, and purulent sputum. Data were expressed as n (%) or median (interquartile range), where appropriate. *p* values were calculated by the Mann-Whitney *U* test, Chi-square test, or Fisher exact test, where appropriate. Abbreviations: COPD, chronic obstructive pulmonary disease; AECOPD, acute exacerbation of COPD; TRQ, tanreqing injection; CAT, the COPD assessment test; mMRC, modified British medical research council; ICU, intensive care unit; HFNC, high-flow nasal cannula oxygen therapy; NPPV, non-invasive positive pressure ventilation; IPPV, invasive positive pressure ventilation.

## Discussion

This was a real-world, national wide, multi-center registry study investigating the efficacy of TQR injection in the treatment of AECOPD Patients. In this study, we found TRQ was effective in lowering CAT score at discharge and ICU admission rate, especially for those with phlegm-heat symptom complex on admission ≥2 and PEACE score >7 on admission among frequent AECOPD patients. For those with infrequent AECOPD, the efficacy is to be further explored. The results of our study provided robust support to the real-world evidence on the clinical use of TRQ and clues for the future investigation of mechanisms of TRQ.

In traditional Chinese medicine, COPD is placed in the same category as cough, dyspnea, and lung distention. Patients with AECOPD often have a series of symptoms of exacerbated cough, increased amounts of sputum, purulent sputum, and fever belonging to the Chinese medicine syndrome of phlegm-heat congestion of the lungs. For such a syndrome, the common treatment principle is clearing the heat and dissipating the phlegm (Li et al., 2010). TRQ consists of Scutellariae radix (SR, *Scutellaria baicalensis* Georgi), bear bile powder (BBP, Selenaretos thibetanus Cuvier), Cornu Caprae Hicus (CCH, Naemorhedus goral Hardwicke), Lonicerae japonicae flos (LJF, *Lonicera japonica* Thunb.), and Forsythiae fructus (FF, *Forsythia suspensa* (Thunb.) Vahl), and it is in accordance with the formulation of Tanreqing injection for treating syndromes including fever, cough, and expectoration (Han et al., 2022).

In our study, TRQ injection was effective in frequent AECOPD inpatients instead of infrequent patients. Frequent and infrequent AECOPD has been recently considered as different phenotypes: patients with frequent exacerbations may have increased airway inflammation in a stable state. More frequent exacerbations were associated with greater impairment in health status, a history of gastroesophageal reflux, and an elevated white-cell count (Hurst et al., 2010).

Modern pharmacological studies have found that Scutellaria baicalensis contained in TRQ injection has antioxidant, free radical scavenging, anti-infection, and antiviral effects; bear gall has anti-infective, sedative, and antispasmodic effects; goat horn has a strong antipyretic effect; honeysuckle contains chlorogenic acid and isochlorogenic acid, which has broad-spectrum antibacterial effect (Jiao et al., 2019). Moreover, laboratory studies showed that effective constituents of TRQ injection promoted the anti-inflammation progress in AECOPD patients. TRQ may improve lung function by inhibiting airway mucus hypersecretion and alleviating airway obstruction and inflammatory injury. Its action pathway may include inhibiting MAPK/NF-κB, which regulates IL-10 and TNF-α release, as well as regulating MUC5AC mRNA expression, which attenuates airway inflammation, airway damage, and mucus hypersecretion (Han et al., 2022). Several clinical studies have suggested that TRQ can regulate cytokines in AECOPD patients, reduce the activation and recruitment of neutrophils and other inflammatory cells in the respiratory tract, slow down the inflammatory process, and promote patient recovery (Yang et al., 2014; Yong et al., 2015). In our study, inpatients with frequent AECOPD were with significantly higher inflammation levels, compared to those with infrequent AECOPD. The drug reaction of TRQ may be influenced by inflammation status, but more studies are needed to verify this finding.

Patients with frequent AECOPD were known for risk factors such as a long time of COPD diagnosis, a larger amount of daily sputum production, higher mMRC score, lower predicted FEV1, and hospitalization during the previous year (Le Rouzic et al., 2018). Our study was consistent with previous findings and also found that inpatients with frequent AECOPD had a higher rate of death, ICU admission, and systemic corticosteroid use, compared with those with infrequent AECOPD. This indicated that among frequent AECOPD patients, antibiotic resistance may be common and inflammation status of the host has been changed for long-term, high-dose corticosteroids as well. As a classic traditional Chinese medicine, TRQ functions in the regulation of homeostasis and rebalance, thus contributing to the recovery of frequent AECOPD, especially for those with more than two phlegm-heat symptoms on admission.

Our study has some limitations. Firstly, we lacked data on biomarkers in our study, making it difficult to analyze the efficacy of TRQ combined with inflammation status. Secondly, the information on the use of antibiotics was missing. Patients with frequent or infrequent AECOPD may be administered with different grades of antibiotics and have different drug resistance statuses, which possibly impacts the course of treatment, length of hospital stay, and clinical outcomes as well. Thirdly, the follow-up data on rehospitalization were temporarily unavailable, therefore we were unable to evaluate the long-term efficacy of TRQ. However, our study revealed that the short-term efficacy was significant and provided important clues for future studies.

## Conclusion

TRQ injection had better efficacy in patients with frequent AECOPD in reducing ICU admission and alleviating respiratory symptoms, especially for those with higher severity on admission or more phlegm-heat symptoms. TRQ may have the potential to improve the long-term prognosis of AECOPD, but more studies are needed to verify this.

## Data Availability

The raw data supporting the conclusion of this article will be made available by the authors, without undue reservation.
