# Supplementary material for: Better response to Tanreqing injection in frequent acute exacerbation of chronic obstructive pulmonary disease (AECOPD) patients—Real-world evidence from a nationwide registry (ACURE) study
Source: Front Pharmacol. 2023 Mar 16;14:1118143. doi: 10.3389/fphar.2023.1118143 (PMC10086601; doi:10.3389/fphar.2023.1118143)
Supplement: Supplementary file 1 [file Table1.docx]

| **Supplementary table 1. Characteristics of inpatients with frequent AECOPD or Infrequent AECOPD after propensity score match** | | | | | | | |  |
| --- | --- | --- | --- | --- | --- | --- | --- | --- |
| **Frequent AECOPD** | | | | | **Infrequent AECOPD** | | | |
| **Characteristics** | **Total** | **TRQ** | **Without TRQ** | **p** | **Total** | **TRQ** | **Without TRQ** | **p** |
|  | **N = 1479** | **N = 493** | **N = 986** |  | **N = 1074** | **N = 358** | **N = 716** |  |
| Age, yrs | 70.8 (65.1, 77.2) | 71.0 (65.1, 77.1) | 70.7 (65.0, 77.3) | 0.8804 | 69.4 (63.0, 75.5) | 69.7 (63.3, 75.6) | 69.2 (62.8, 75.4) | 0.5110 |
| Male | 1179 (79.7) | 388 (78.7) | 791 (80.2) | 0.4928 | 779 (72.5) | 272 (76.0) | 507 (70.8) | 0.0737 |
| BMI, Kg/m^2^ | 21.6 (19.2, 24.2) | 21.8 (19.5, 24.2) | 21.6 (19.1, 24.2) | 0.3861 | 22.0 (19.6, 24.2) | 22.0 (19.5, 24.2) | 22.0 (19.6, 24.3) | 0.4645 |
| Smoking |  |  |  | 0.7208 |  |  |  | 0.8997 |
| Current smoking | 265 (17.9) | 91 (18.5) | 174 (17.6) |  | 306 (28.5) | 102 (28.5) | 204 (28.5) |  |
| Never smoking | 463 (31.3) | 159 (32.3) | 304 (30.8) |  | 366 (34.1) | 119 (33.2) | 247 (34.5) |  |
| Quit smoking | 751 (50.8) | 243 (49.3) | 508 (51.5) |  | 402 (37.4) | 137 (38.3) | 265 (37.0) |  |
| Pulmonary thromboembolism | 8 (0.5) | 3 (0.6) | 5 (0.5) | 0.8039 | 1 (0.1) | 0 (0.0) | 1 (0.1) | 0.3677 |
| Pulmonary artery hypertension | 69 (4.7) | 25 (5.1) | 44 (4.5) | 0.6009 | 24 (2.2) | 8 (2.2) | 16 (2.2) | 1.0000 |
| Hypertension | 477 (32.3) | 154 (31.2) | 323 (32.8) | 0.5552 | 333 (31.0) | 103 (28.8) | 230 (32.1) | 0.2629 |
| Myocardial infarction | 245 (16.6) | 78 (15.8) | 167 (16.9) | 0.5864 | 142 (13.2) | 42 (11.7) | 100 (14.0) | 0.3081 |
| Cor pulmonale | 399 (27.0) | 133 (27.0) | 266 (27.0) | 1.0000 | 153 (14.2) | 50 (14.0) | 103 (14.4) | 0.8531 |
| Bronchiectasia | 131 (8.9) | 50 (10.1) | 81 (8.2) | 0.2189 | 69 (6.4) | 24 (6.7) | 45 (6.3) | 0.7918 |
| Non-drug therapy | 796 (53.8) | 259 (52.5) | 537 (54.5) | 0.4835 | 266 (24.8) | 89 (24.9) | 177 (24.7) | 0.9601 |
| Drug therapy | 1021 (69.0) | 338 (68.6) | 683 (69.3) | 0.7807 | 396 (36.9) | 132 (36.9) | 264 (36.9) | 1.0000 |
| Regular inhaled corticosteroid | 68/128 (53.1) | 32/56 (57.1) | 36/72 (50.0) | 0.4218 | 14/29 (48.3) | 3/8 (37.5) | 11/21 (52.4) | 0.4715 |
| Regular oral corticosteroid | 16/69 (23.2) | 8/31 (25.8) | 8/38 (21.1) | 0.6416 | 5/24 (20.8) | 4/11 (36.4) | 1/13 (7.7) | 0.0787 |
| Inhaled corticosteroids | 128 (8.7) | 56 (11.4) | 72 (7.3) | 0.0089 | 29 (2.7) | 8 (2.2) | 21 (2.9) | 0.5057 |
| Inhaled bronchial dilator | 305 (20.6) | 122 (24.7) | 183 (18.6) | 0.0056 | 105 (9.8) | 36 (10.1) | 69 (9.6) | 0.8275 |
| PEACE at admission | 7.0 (6.0, 9.0) | 8.0 (6.0, 9.0) | 7.0 (6.0, 9.0) | 0.7580 | 7.0 (6.0, 9.0) | 7.0 (6.0, 9.0) | 7.0 (6.0, 9.0) | 0.9586 |
| CAT at admission | 21.0 (16.0, 26.0) | 21.0 (17.0, 25.0) | 21.0 (16.0, 26.0) | 0.6107 | 19.0 (14.0, 24.0) | 19.0 (15.0, 24.0) | 19.0 (14.0, 24.0) | 0.8524 |
| mMRC at admission | 3.0 (2.0, 3.0) | 3.0 (2.0, 3.0) | 3.0 (2.0, 3.0) | 0.8834 | 3.0 (2.0, 3.0) | 3.0 (2.0, 3.0) | 3.0 (2.0, 3.0) | 0.9058 |
| Diagnosed as COPD for the first time | 90 (6.1) | 21 (4.3) | 69 (7.0) | 0.0378 | 614 (57.2) | 187 (52.2) | 427 (59.6) | 0.0208 |
| Hospitalization frequency due to AECOPD | 2.0 (1.0, 2.0) | 2.0 (1.0, 2.0) | 2.0 (1.0, 2.0) | 0.9957 | 0.0 (0.0, 0.0) | 0.0 (0.0, 0.0) | 0.0 (0.0, 0.0) | 1.0000 |
| Cough frequency |  |  |  | 0.1049 |  |  |  | 0.5889 |
| All day | 747 (50.5) | 237 (48.1) | 510 (51.7) |  | 550 (51.2) | 193 (53.9) | 357 (49.9) |  |
| Continuously | 185 (12.5) | 68 (13.8) | 117 (11.9) |  | 163 (15.2) | 50 (14.0) | 113 (15.8) |  |
| No cough | 17 (1.1) | 2 (0.4) | 15 (1.5) |  | 12 (1.1) | 3 (0.8) | 9 (1.3) |  |
| Occasionally | 530 (35.8) | 186 (37.7) | 344 (34.9) |  | 349 (32.5) | 112 (31.3) | 237 (33.1) |  |
| Amount of sputum |  |  |  | 0.8821 |  |  |  | 0.9655 |
| < 50 ml | 836 (56.5) | 280 (56.8) | 556 (56.4) |  | 565 (52.6) | 188 (52.5) | 377 (52.7) |  |
| ≥ 50 ml | 643 (43.5) | 213 (43.2) | 430 (43.6) |  | 509 (47.4) | 170 (47.5) | 339 (47.3) |  |
| Purulent sputum | 744 (50.3) | 250 (50.7) | 494 (50.1) | 0.8254 | 495 (46.1) | 153 (42.7) | 342 (47.8) | 0.1192 |
| Fever | 270 (18.3) | 92 (18.7) | 178 (18.1) | 0.7752 | 179 (16.7) | 61 (17.0) | 118 (16.5) | 0.8169 |
| Pharyngalgia | 143 (9.7) | 46 (9.3) | 97 (9.8) | 0.7557 | 83 (7.7) | 23 (6.4) | 60 (8.4) | 0.2580 |
| PH | 7.4 (7.4, 7.4) | 7.4 (7.4, 7.4) | 7.4 (7.4, 7.4) | 0.0042 | 7.4 (7.4, 7.4) | 7.4 (7.4, 7.4) | 7.4 (7.4, 7.4) | 0.4701 |
| PACO2 | 43.9 (38.2, 52.0) | 43.0 (38.1, 51.0) | 44.0 (38.3, 53.3) | 0.2173 | 42.0 (37.8, 48.3) | 42.0 (37.9, 48.0) | 42.0 (37.6, 48.7) | 0.9949 |
| PAO2 | 73.0 (60.0, 89.6) | 75.0 (61.0, 94.0) | 72.0 (59.9, 87.0) | 0.0065 | 72.8 (63.0, 87.0) | 74.0 (64.5, 88.5) | 72.0 (62.0, 86.3) | 0.1501 |
| High sensitivity C reactive protein, mg/dl | 5.0 (1.0, 16.0) | 4.3 (0.8, 13.1) | 5.0 (1.2, 18.6) | 0.0267 | 3.6 (0.8, 12.0) | 2.6 (0.6, 10.0) | 4.0 (1.0, 12.3) | 0.0902 |
| ≥3, mg/dl | 549/908 (60.5) | 181/325 (55.7) | 368/583 (63.1) | 0.0282 | 356/675 (52.7) | 110/227 (48.5) | 246/448 (54.9) | 0.1126 |
| PCT, ng/ml | 0.1 (0.0, 0.2) | 0.1 (0.0, 0.1) | 0.1 (0.0, 0.2) | 0.7226 | 0.1 (0.0, 0.1) | 0.1 (0.0, 0.1) | 0.1 (0.0, 0.1) | 0.1818 |
| ≥0.1, ng/ml | 354/887 (39.9) | 125/308 (40.6) | 229/579 (39.6) | 0.7648 | 259/669 (38.7) | 75/222 (33.8) | 184/447 (41.2) | 0.0650 |
| White blood cell count, × 10^9^/L | 7.4 (5.7, 9.9) | 7.5 (5.8, 9.8) | 7.3 (5.7, 10.0) | 0.3592 | 7.3 (5.7, 9.3) | 7.1 (5.8, 9.2) | 7.3 (5.7, 9.3) | 0.5756 |
| Neutrophils, % | 72.0 (63.0, 80.4) | 72.9 (63.1, 80.9) | 71.8 (62.9, 80.3) | 0.4937 | 70.2 (60.9, 79.1) | 70.5 (60.4, 78.5) | 70.1 (61.1, 79.5) | 0.6325 |
| Lymphocyte, % | 16.8 (10.0, 24.0) | 16.5 (9.8, 23.8) | 17.0 (10.1, 24.0) | 0.5060 | 18.1 (10.7, 26.5) | 18.1 (11.2, 26.9) | 18.1 (10.4, 26.3) | 0.9655 |
| Aspartate aminotransferase, U/L | 19.2 (15.3, 26.0) | 19.2 (15.2, 26.4) | 19.2 (15.3, 26.0) | 0.7107 | 19.8 (15.3, 26.0) | 19.0 (15.0, 24.0) | 20.0 (16.0, 27.0) | 0.0116 |
| > 40, U/L | 117/1416 (8.3) | 46/483 (9.5) | 71/933 (7.6) | 0.2149 | 73/1035 (7.1) | 18/347 (5.2) | 55/688 (8.0) | 0.0959 |

Note. Data were expressed as n (%) or median (interquartile range), where appropriate. P values were calculated by Mann-Whitney U test, Chi-square test, or Fisher exact test, where appropriate. Abbreviations: COPD, chronic obstructive pulmonary disease; AECOPD, acute exacerbation of COPD; TRQ, Tanreqing injection; BMI, body mass index; CAT, the COPD assessment test; mMRC, modified British medical research council; PCT, procalcitonin.

| **Supplementary table 2. Treatment and clinical outcomes of frequent AECOPD inpatients with phlegm-heat symptoms ≥1 or no phlegm-heat symptom complex** | | | | | | | | |
| --- | --- | --- | --- | --- | --- | --- | --- | --- |
| **Phlegm-heat symptom complex on admission ≥1** | | | | | **<1** | | | |
| **Characteristics** | **Total** | **TRQ** | **Without TRQ** | **p** | **Total** | **TRQ** | **Without TRQ** | **p** |
|  | **N = 1065** | **N = 355** | **N = 710** |  | **N = 384** | **N = 128** | **N = 256** |  |
| PEACE at discharge | 3.0 (2.0, 4.0) | 3.0 (2.0, 4.0) | 3.0 (2.0, 4.0) | 0.6214 | 3.0 (2.0, 4.0) | 3.0 (2.0, 4.0) | 3.0 (2.0, 4.0) | 0.389 |
| CAT at discharge | 12.0 (9.0, 17.0) | 12.0 (8.0, 17.0) | 13.0 (9.0, 17.0) | 0.1333 | 13.0 (9.0, 17.0) | 12.0 (8.5, 16.0) | 13.0 (9.0, 19.0) | 0.0438 |
| PEACE difference | -5.0 (-6.0, -3.0) | -5.0 (-6.0, -3.0) | -5.0 (-6.0, -3.0) | 0.2828 | -3.0 (-4.0, -2.0) | -3.0 (-4.0, -2.0) | -3.0 (-4.0, -2.0) | 0.454 |
| mMRC at discharge | 2.0 (1.0, 2.0) | 2.0 (1.0, 2.0) | 2.0 (1.0, 2.0) | 0.5061 | 2.0 (1.0, 2.0) | 2.0 (1.0, 2.0) | 2.0 (1.0, 3.0) | 0.1084 |
| mMRC difference | -1.0 (-2.0, 0.0) | -1.0 (-2.0, 0.0) | -1.0 (-2.0, 0.0) | 0.6198 | -1.0 (-1.0, 0.0) | -1.0 (-2.0, 0.0) | -1.0 (-1.0, 0.0) | 0.3445 |
| Length of hospital stay | 11.0 (9.0, 14.0) | 11.0 (9.0, 14.0) | 11.0 (8.0, 14.0) | 0.0271 | 11.0 (9.0, 13.5) | 11.0 (9.0, 13.5) | 10.0 (8.0, 13.5) | 0.3243 |
| Total cost of hospitalization (USD) | 1516.2 (1094.9, 2140.3) | 1517.4 (1061.8, 2074.8) | 1514.6 (1106.7, 2187.8) | 0.2914 | 9626.1 (7181.4, 13643.2) | 8936.2 (6943.2, 12355.5) | 10134.0 (7297.4, 14728.9) | 0.0678 |
| Death or worsen in hospital | 7 (0.7) | 2 (0.6) | 5 (0.7) | 0.7859 | 2 (0.5) | 0 (0.0) | 2 (0.8) | 0.2021 |
| ICU admission | 12/1064 (1.1) | 2/354 (0.6) | 10/710 (1.4) | 0.1928 | 8 (2.1) | 1 (0.8) | 7 (2.7) | 0.1705 |
| Oxygen support |  |  |  | 0.9969 |  |  |  | 0.1871 |
| No oxygen support | 121 (11.4) | 40 (11.3) | 81 (11.4) |  | 42 (10.9) | 16 (12.5) | 26 (10.2) |  |
| Tube/mask | 836 (78.5) | 278 (78.3) | 558 (78.6) |  | 277 (72.1) | 96 (75.0) | 181 (70.7) |  |
| HFNC | 7 (0.7) | 2 (0.6) | 5 (0.7) |  | 6 (1.6) | 2 (1.6) | 4 (1.6) |  |
| NPPV | 98 (9.2) | 34 (9.6) | 64 (9.0) |  | 58 (15.1) | 13 (10.2) | 45 (17.6) |  |
| IPPV | 3 (0.3) | 1 (0.3) | 2 (0.3) |  | 1 (0.3) | 1 (0.8) | 0 (0.0) |  |
| Antibiotics | 998/1064 (93.8) | 339/355 (95.5) | 659/709 (92.9) | 0.1046 | 340 (88.5) | 119 (93.0) | 221 (86.3) | 0.0541 |
| Corticosteroid | 854/1064 (80.3) | 280/355 (78.9) | 574/709 (81.0) | 0.4202 | 317 (82.6) | 105 (82.0) | 212 (82.8) | 0.8492 |

Note. The phlegm-heat symptom complex include fever, pharyngalgia, expectoration, purulent sputum. Data were expressed as n (%) or median (interquartile range), where appropriate. P values were calculated by Mann-Whitney U test, Chi-square test, or Fisher exact test, where appropriate.

Abbreviations: COPD, chronic obstructive pulmonary disease; AECOPD, acute exacerbation of COPD; TRQ, Tanreqing injection; CAT, the COPD assessment test; mMRC, modified British medical research council; ICU, intensive care unit; HFNC, high-flow nasal cannula oxygen therapy; NPPV, non-invasive positive pressure ventilation; IPPV, invasive positive pressure ventilation.
